# Assessment of the Minimum Sampling Frequency to Avoid Measurement Redundancy in Microclimate Field Surveys in Museum Buildings

**DOI:** 10.3390/s16081291

**Published:** 2016-08-15

**Authors:** Fernando-Juan García-Diego, Elena Verticchio, Pedro Beltrán, Anna Maria Siani

**Affiliations:** 1 Department of Applied Physics, Universitat Politècnica de València, Av. de los Naranjos s/n, Valencia 46022, Spain; pbeltran@fis.upv.es; 2 Centro de Tecnologías Físicas, Universitat Politècnica de València, Av. de los Naranjos s/n, Valencia 46022, Spain; 3 Department of Physics, Sapienza Università di Roma, P.le A. Moro 2, Rome 00185, Italy; verticchio.1391542@studenti.uniroma1.it (E.V.); annamaria.siani@uniroma1.it (A.M.S.)

**Keywords:** temperature and relative humidity, sampling rate, museum measurements, indoor microclimate, Sorolla paintings, Pio V Museum

## Abstract

Monitoring temperature and relative humidity of the environment to which artefacts are exposed is fundamental in preventive conservation studies. The common approach in setting measuring instruments is the choice of a high sampling rate to detect short fluctuations and increase the accuracy of statistical analysis. However, in recent cultural heritage standards the evaluation of variability is based on moving average and short fluctuations and therefore massive acquisition of data in slowly-changing indoor environments could end up being redundant. In this research, the sampling frequency to set a datalogger in a museum room and inside a microclimate frame is investigated by comparing the outcomes obtained from datasheets associated with different sampling conditions. Thermo-hygrometric data collected in the Sorolla room of the Pio V Museum of Valencia (Spain) were used and the widely consulted recommendations issued in UNI 10829:1999 and EN 15757:2010 standards and in the American Society of Heating, Air-Conditioning and Refrigerating Engineers (ASHRAE) guidelines were applied. Hourly sampling proved effective in obtaining highly reliable results. Furthermore, it was found that in some instances daily means of data sampled every hour can lead to the same conclusions as those of high frequency. This allows us to improve data logging design and manageability of the resulting datasheets.

## 1. Introduction

Interest in the preservation of cultural heritage is the consequence of its intrinsic sensitivity to the environment. Preventive conservation is based on the idea that it is possible to guarantee the durability of artworks by controlling some of the main causes of deterioration and focuses on avoiding the need for further restoration interventions [1]. Monitoring of the main microclimatic parameters, such as temperature (T) and relative humidity (RH), is fundamental to verify whether the ambient conditions are suitable for conservation purposes and allows us to investigate possible sources of degradation and suggest mitigating measures [2].

The common approach in setting the measuring instruments in a monitoring system consists of choosing a high sampling frequency (for instance, one measurement per minute), in order to increase the accuracy on the estimate of statistical time descriptors (e.g., the hourly mean) and to detect up to the shortest-term environmental fluctuations. A high frequency acquisition, however, involves large volume data storage and high computing power to process and elaborate data. In the definition of the time interval between consecutive measurements, the response time of the materials is a key factor to be taken into account [3,4], as materials do not respond immediately to changes in the environment and fluctuations with a period inferior to one hour do not affect most museum objects [5]. Furthermore, in recent standards on microclimate the assessment of seasonal variability based on the statistical tool of moving average and massive acquisition of data in slowly-fluctuating indoor environments could end up being redundant. Collecting data with high sampling frequency (e.g., one minute) can be inadequate, both in terms of the memory capacity needed and the scarce manageability of the resulting file. In fact, common software suites for handling data (e.g., Microsoft Excel or LibreOffice) are unable to deal with very large data sets and this can be a special problem for conservators having limited ability to undertake computations with large amounts of collected data. In recent years, many remarkable efforts have been made in the field of monitoring design, exploiting machine learning to increase the efficiency in remote control, and dataloggers’ management [6,7]. Computational intelligence can also be extremely helpful in implementing low-cost tools for suitably improving sampling conditions [8,9], but preliminary surveys based on this approach are still rarely employed to configure museum monitoring systems. The advantages and disadvantages of the recent standards in cultural heritage conservation have already been discussed in some recent papers [10,11,12,13,14,15], but none of these works has involved a critical examination of the choice of sampling frequency.

In many countries, such as Spain, environmental standards for cultural heritage collections are not available. Guidelines and recommendations for preventive conservation are thus generally based on foreign standards [16], among which the most widely consulted are the Italian UNI 10829:1999, the European EN 15757:2010 and the ASHRAE guidelines (2011). Italian regulation UNI 10829:1999 [17] specifies the hygrothermal ranges recommended for different typologies of materials and the majority of Italian and worldwide museums has adopted it for indoor climate control. This standard recommends that analysis of the microclimatic quantities monitored should be based on measurements sampled at 1 h for a period sufficiently long to allow the understanding of the temporal trends. European standard EN 15757:2010 [18] defines the historical climate, i.e., climatic conditions to which an object has resisted for a long period under reasonably acceptable conditions and to which it has acclimatized [19]. This standard recommends that the historical climate be maintained, especially as far as RH is concerned if the object has been found in good conditions. Sampling intervals should be one hour or less, in order to respond to the time scale and the dynamics of the phenomena under investigation. Recent studies have applied the above regulation to reconstruct past indoor climates and to assess the impact of the expected future climate change [19,20]. In the ASHRAE (American Society of Heating, Air-Conditioning and Refrigerating Engineers) guidelines [21], five classes of quality control are defined on the basis of seasonal and daily T and RH fluctuations. The possible risk for collections is given for each class: class AA is associated with no risk to most objects; class A with low risk to highly vulnerable objects (e.g., those made of organic hygroscopic materials) and no risk to most objects; Class B with moderate risk to highly vulnerable objects and low risk to most objects; Class C is able to prevent only high risk extremes and class D can protect only from dampness. Class A is divided into two subclasses having the same level of risk, which were called as Class A and As by Martens in [22]: ‘As’, with seasonal adjustments but smaller daily fluctuations and ‘A’, with larger daily fluctuations but no seasonal adjustment. In these guidelines there is no reference to the use of a specific sampling frequency. The guidelines are further discussed in [22,23] and have been recently applied to assess the damage risk of future climate scenarios [24]. 

The aim of the present research is to investigate whether there are differences in the applications of standards using datasets with different sampling rates and hence to determine the minimum sampling frequency to set a datalogger in microclimate field surveys in museum buildings. The results obtained by applying common conservation standards to datasheets of different sampling conditions were compared. For this purpose we analyzed the large microclimatic data collection recorded at 1 min in step resolution in the Sorolla Room of the Museum of Fine Arts of Valencia. Notwithstanding that in our study a specific case was considered, however in the application of cultural heritage conservation standards the detection of the sampling rate that has minimum frequency and produces the same outcome as those of high frequency can be highly effective in improving sampling design and in handling the resulting datasheet. A preliminary exploratory analysis on data collected provided the assessment of the general performance of the microclimate frames in relation to the environment of the room of the museum.

## 2. Materials and Methods

### 2.1. The Sorolla Paintings

**Figure 1:**
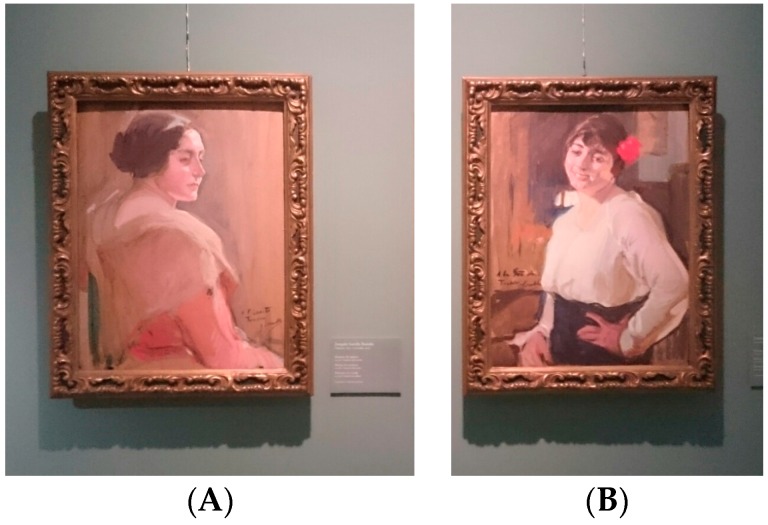
Portraits by Joaquín Sorolla (1863–1923), painted in 1916 with the technique of gouache on cardboard. Painting (**A**), on the left, is titled “Portrait of Madame” and painting (**B**), on the right, is “Portrait of a lady with a red flower in her hair”.

A monitoring campaign was conducted in the Sorolla Room of the Pio V Museum of Fine Arts of Valencia (Spain) to study the conservation environment of two paintings by Joaquín Sorolla (1863–1923). The portraits, titled “Portrait of a lady with a red flower in her hair” and “Portrait of Madame” (Figure 1), were painted in 1916 with the technique of gouache on cardboard and are the property of the Traver family. The pieces used to be conserved in a private house and kept in frames whose glass front was in contact with the picture, so that a problem of degradation emerged. In fact, although the growth of fungi is unlikely related to high frequency RH changes, conservation surveys performed by the Valencian Institute of Conservation and Restoration (IVACOR) highlighted that uncontrolled levels of relative humidity, together with the sensitivity of the materials used for the technique of gouache on cardboard, could have stimulated the growth of fungi responsible for some stains observed on the paintings [25].

The artworks underwent restoration from 2012 to 2014 in the IVACOR and at the end of the intervention were enclosed in microclimatic ad hoc designed frames [25]. From April 2014 to February 2016 they were on display, with 54 other works by the artist, in the Sorolla Room of the Museum of Fine Arts of Valencia [26], where an active HVAC (Heating Ventilating and Air-Conditioning) system of microclimatic control was in operation. Showcases are frequently employed in museums as preventive conservation means to protect artefacts by reducing the risks linked to dust and accidental damage as well as offering an effective protection from the environment to which objects are exposed [27,28,29]. A microclimate frame is a specifically designed frame for controlling the thermo-hygrometric indoor conditions and is nowadays considered the safest system for keeping relative humidity stable in the case of paintings sensitive to fluctuations [30]. A buffering agent (e.g., silica gel and other hygroscopic materials) is usually added to decrease fluctuations of RH values driven by temperature changes (e.g., due to external forcing such as HVAC systems, lighting and visitors) [27,28], particularly in those vitrines not perfectly sealed [31]. Continuously recording T and RH values within and outside the frame is thus fundamental to assess the buffering performances of the microclimate frames over time [28]. After the restoration, a T and RH monitoring system developed at the Department of Applied Physics of the UPV (Polytechnic University of Valencia) [32] was installed. The thermo-hygrometers were positioned both inside and outside the frames and set to sample the temperature and relative humidity values with the sampling frequency of one minute.

The portraits by Sorolla measure 64 cm × 49 cm and are enclosed in handcrafted airtight microclimatic frames (69 cm × 54 cm × 8 cm) made of an external aluminum case and a display glass. A sheet of cardboard (the same material used as support for the portraits) with the same dimensions of the paintings was used as back plate for the frames. The cardboard is in direct contact with the painting to offset changes in external relative humidity, acting as a buffering agent. In the museum, the paintings were positioned on the same wall and at the same height, separated by a distance of 1 m, as shown in Figure 1.

### 2.2. Microclimate Monitoring Campaign

**Figure 2:**
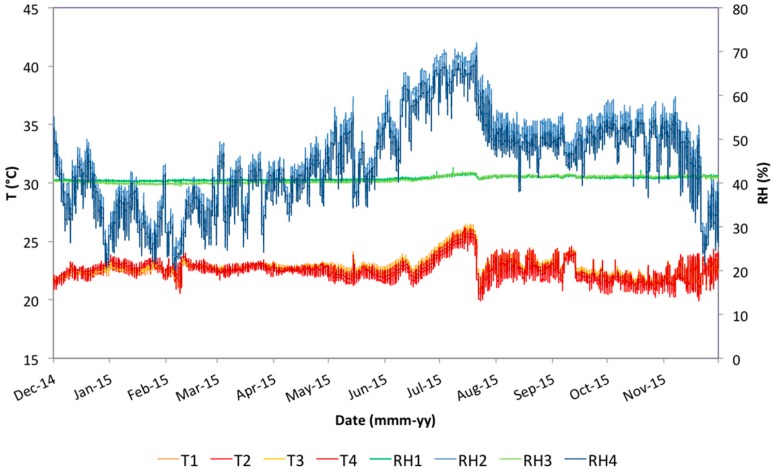
Temperature (T) and relative humidity (RH) values collected by the probes during a year of monitoring, from 1 December 2014 to 30 November 2015 (probes 1 and 2 representing the environment inside and outside of the microclimatic frames of Painting **A** and probes 3 and 4 representing the same for Painting **B**).

Monitoring of the portraits during the exhibition at the Pio V Museum started on 1 May 2014, after the restoration intervention, and finished on 13 February 2016. The sampling frequency was set to 60 acquisitions per hour (one measurement every minute). Thermo-hygrometric data studied in this paper cover the period from 1 December 2014 to 30 November 2015 (Figure 2), as the application of the different standards requires analysis lasting at least one whole year. The time series were then extended by one or three months more where necessary for calculation of the moving averages. On 13 February the paintings were removed, together with the probes, from the museum and the same sensors are still monitoring them.

The monitoring system was developed at the Department of Applied Physics of the UPV [32]. Four probes, each with a temperature sensor and one of relative humidity, were assembled and positioned within and outside each microclimate frame. Power supply and data cables were hidden in the hanging systems of frames used in the museum (Figure 1). Table 1 shows the position of probes in the monitoring campaign.

Air temperature was measured with a digital thermometer (accuracy: ±0.5 °C in the range of measurement from −10 °C to +85 °C; temperature conversion time: 750 ms); relative humidity was measured with a capacitive hygrometer (accuracy: ±3.5% in the range of measure from 10% to 95%; response time 5 s). Calibration of RH sensors with aqueous solutions of two salts (lithium chloride and sodium chloride) was performed according to the ASTME 104-02 [33] standard to reduce measurement error. The technical features of temperature sensors [34] are in accordance with instrumental metrological characteristics recommended in EN 15758:2010 [35], while the accuracy of relative humidity sensors [36] is slightly below the requirement of 3% issued by EN 16242:2012 [37].

### 2.3. The Application of Standards and Guidelines 

The standards were addressed in the following order: the Italian regulation of 1999 (UNI 10829:1999), which is still in use in most Italian and worldwide museums, the European standard of 2010 (EN 15757:2010) and finally the American guidelines (ASHRAE) of 1999, republished in 2011 ASHRAE Handbook. The UNI 10829:1999 standard [17] specifies ranges of T and RH tolerated for each typology of material and the risks for conservation linked to the environment can be assessed, calculating the percentage of time in which the measured values are maintained in the range acceptable. For application of the standard, the values indicated for the conservation of paper were used, as this material constitutes the support of the portraits. For paper, the regulation states that the best intervals are the range from 18 to 22 °C for temperature and the range from 40% to 55% for relative humidity. It also recommends that the maximum daily variations should be of 1.5 °C for T and of 6% for RH values. EN 15757:2010 [18] proposes a method to establish the historic microclimate based on a 30 days central moving average (MA) of the data collected, through which the environmental seasonal variability is assessed. In addition to the seasonal trend, it is also necessary to evaluate the short-term fluctuations, which exert a great influence on the response of the collections. As established by the standard, the short term fluctuations are calculated as the difference between the measured values and seasonal cycle, thus it is possible that the sampling frequency of the registers may have some influence on the application of the standard. The bands of tolerable variations for relative humidity are obtained superimposing on the MA the lower and the upper limits, calculated as the 7th and the 93rd percentiles of the distribution of the short-term fluctuations MA value, respectively. Each pair of bands of tolerance was approached separately, i.e., using data with different sampling frequencies. The ASHRAE guidelines of 2011 [21] are based on a central moving average of 91 days. The classification in classes of environmental quality is based both on seasonal adjustments and short fluctuations. The maximum and minimum seasonal shift is calculated by adding and subtracting to the annual mean the seasonal changes allowed for each class. If the calculated moving average is below the minimum or above the maximum allowed value, the average value is replaced by this limiting value [22]. The width of the final bands is finally determined shifting the moving average curve by the short-term fluctuations indicated for each class. A website is also available to easily obtain graphs from climate data series [38].

### 2.4. Dataset Sampling Frequency

All the following elaborations were developed implementing scripts in R programming language [39]. Different data logging conditions were simulated by the extraction of the first data over intervals of 15 min, 30 min, and one hour, and daily means of the microclimatic parameters were calculated from data sampled every hour. Following this procedure, the original file dimensions could be significantly reduced and new datasheets were created to be elaborated according to the recommendations of the standards. For the comparison of the results, we chose to adopt the Performance Index (PI), defined as the percentage of time for which the measured environmental parameter lies inside the recommended range [40]:

$$PI\;\left[\%\right]=\left(\frac{\sum \tau_{\left(t\;within\;tolerance\;range\right)}}{\sum_{0}^{total\;occupancy\;time}\tau }\right)\cdot \%$$

The use of a single index is considered as essential, at least at the first stage of the analysis, since raw datasets are too long to analyze and difficult to express in terms of adherence to the standards’ recommendations [41]. However, it has to be pointed out that the PI does not provide a direct assessment of the preservation risks for the artifacts. By means of this synthetic indicator, the outcomes obtained from the datasets associated with different sampling conditions can be easily compared to the reference results of one-minute data.

## 3. Results and Discussion

### 3.1. Exploratory Data Analysis

The exploratory analysis was carried out using data collected with 1 min sampling rate by the four probes. Figure 2 shows the trends of the thermo-hygrometric values collected every minute by the four probes, from 1 December 2014 to 30 November 2015. As we can see in Figure 2, the time series considered covers a period of nearly two months (from the end of May until the end of July 2015) during which the air-conditioning system of the museum stopped working, allowing temperature and relative humidity values to increase [42]. However, that time interval was also taken into account in the analysis to verify how an evident condition of danger for conservation is evaluated by the different standards in use.

**Figure 3:**
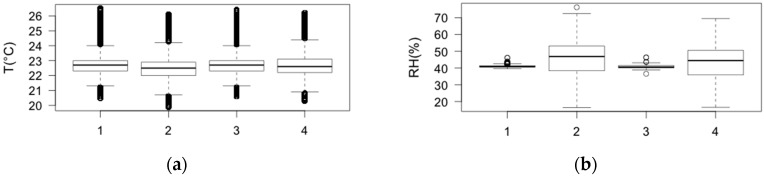
Box-and-whisker plot of T (**a**) and RH (**b**) inside (probes 1 and 3) and outside (probes 2 and 4) the microclimate frames. The outliers, i.e., the values above or below 1.5 × IQR (IQR = interquartile range), are indicated with a circle.

The Wilcoxon-Mann-Whitney test was used to establish significant differences among T and RH data retrieved by the four probes. This test assumes the samples are not normally distributed. The significance level was set to 5%. The test was performed for each pair of T and RH sensors. No significant difference was found among temperature series (*p* > 0.05). Indeed it was found that both internal RH data sets are significant lower (*p* < 0.0001) than those outside the frames, whereas both internal and external RH data set pairs (RH1 with RH3 and RH2 with RH4, respectively), do not show significant differences. The Box-and-Whiskers plots are used for a synthetic visualization of data (Figure 3a,b).

From the box plots of temperature (Figure 3a) it is evident that there is no significant difference among data collected throughout the year by the four probes, as the medians and the boxes fully overlap. The mean value of T medians is 22.6 °C and the variability is less than ±2 °C for all the sensors. Most of the outliers found in T data are caused by the problem that occurred with the air conditioning system and, as they are associated with the real microclimate of the room, they were considered in the following analysis. The box plots of RH in Figure 3b show that the values registered inside the microclimate frame (probes 1 and 3) and the relative humidity of the rest of the room (probes 2 and 4) are not correlated. The RH median related to inside sensors is 40.8%, with values ranging between 38.8% and 43.2%. The mean value of RH medians of the outside sensors is 45.7%, with variability between 16.5% and 72.5% (25th and 75th percentiles of the distribution, respectively). The outliers found for the RH data are associated with high local values (probably due to instantaneous increases in the air moisture content) and not discarded in the analysis.

**Figure 4:**
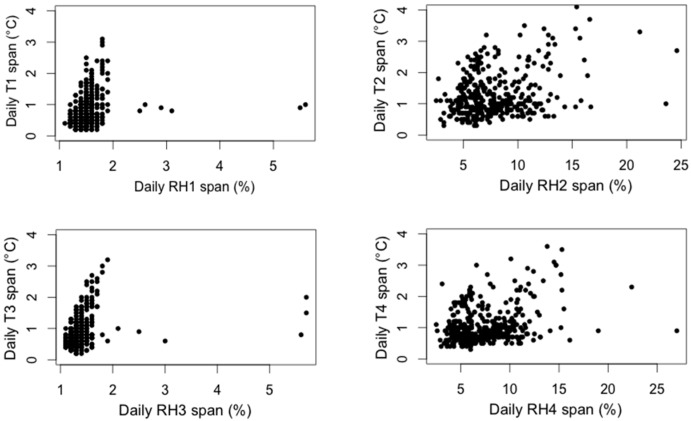
Scatter diagram of daily RH span versus daily T span inside (probes 1 and 3) and outside (probes 2 and 4) the microclimate frames. The daily span is calculated as the difference between the maximum and minimum values of each day.

RH daily span versus T daily span for the four sensors are shown in Figure 4. The daily span is defined as the difference between the maximum (max) and the minimum (min) value observed within each day and its calculation is useful to study the variability of the parameters throughout the year.

The behavior of the two groups of sensors can be easily distinguished in the graphs in Figure 4: as expected, data from probes inside the microclimate frames (probes 1 and 3) show less variability in temperature and are very stable in terms of relative humidity, in fact the small RH changes (typically 1%–2%) are below the range of the instrumental accuracy (3.5%). The values collected in the room (probes 2 and 4) are associated with strong daily fluctuations of relative humidity. In the case of the probes installed in the room, the maximum variation of T during the same day is around 4 °C for probe 2 and around 3.5 °C for probe 4, but RH daily spans can exceed 20%. In few cases large RH daily spans occurred, although a physical interpretation was not found because the causes are still unknown. However, since they were not due to instrumental problems, they were included in the analysis. 

In Table 2 the daily cycles of T and RH data collected from each probe are summarized in terms of average, maximum, and mode of the daily T and RH spans.

According to Michalski [5], paper’s response time to RH changes lies between minutes and hours, depending also on the thickness of the sheet [22]. Hence, these variations can be very dangerous for organic hygroscopic materials and it is likely that they could have induced mechanical damage to the portraits if microclimatic frames had not been provided after the restoration.

### 3.2. Italian Regulation UNI 10829:1999

The Performance Index was calculated as the percentages of time in which the thermo-hygrometric parameters were in the acceptable range for paper [17]. The PI values obtained from the reference data of one-minute sampling were compared with those resulting from data extracted every 15 min, every 30 min, and every hour.

Table 3 summarizes the outcomes obtained from data collected at the frequency of one minute.

The outcomes show that the PI_MINUTE_ values for collected T data are very small both inside and outside the microclimate frames. In the room environment, just half of the RH data registered can be considered adequate according to the standard. Inside the microclimate frames, the temperature was found slightly worsened compared to the room, probably because of the boxes that act as an insulator for heat exchanges. On the contrary, internal extreme RH values are cut off by the presence of the buffer, as expected. The different behavior of the two microclimate frames is just apparent, as the lower number of occurrences inside the range of probe 3 is due to deviations from the limits that are not significant on the basis of the error associated with the initial RH value imposed inside the frame. In fact, the restorers decided to set the paintings an RH of 40% (the limit of the range recommended in the standard) and every little deviation from this value led to RH out of the lower limit.

Table 4 shows the differences of the PI of the simulated frequencies from the PI_MINUTE (range)_ reference. 

The outcomes show that the percentages of T and RH values that fall inside the acceptable ranges are independent of sampling frequency. The hourly measurements recommended by the standard thus provide results identical to those obtained with one-minute frequency.

Table 5 shows the percentages of T and RH daily variations below the tolerated limit for paper for the reference one-minute dataset (PI_MINUTE(daily span)_). Average, maximum and mode of the daily T and RH spans in the year considered are shown in Table 4.

The PI values shown in Table 4 indicate that the daily variations of temperature in the museum room respect the limits in 74% of cases for sensor 2 and 82% for sensor 4, while the changes in RH values within the same day fall in the interval recommended only in 30% and 40%, respectively. The thermo-hygrometric variations inside the microclimate frames are definitely safer than those of the room and can be considered ideal for the conservation of paper (except for approximately 10% of T daily spans that exceed the limit recommended). In fact, thanks to the RH buffering action—exerted by the painting itself and by the buffering agent—and to the delay with which they respond to the external T changes, the microclimate frames proved to be able to cut off the major daily fluctuations.

Table 6 shows the differences between the PI values associated with the simulated sampling conditions and the PI_MINUTE(daily span)_ reference (ΔPI_15Min_, ΔPI_30Min_, and ΔPI_Hour_).

The differences between the PI obtained for daily spans (Table 5) appear to be significant in the case of the outside probes (2 and 4), so the reduction of the recording frequency may not be valid when the purpose is to evaluate daily fluctuations. When the variation is small in amplitude, as for probes 1 and 3, the sampling frequency affects the outcomes less. However, this result seems to be strongly affected by the randomness associated with the data acquisition method, as the major fluctuations appear to be smoothed proportional to the extent of the interval considered.

### 3.3. European Standard EN 15757:2010

**Figure 5:**
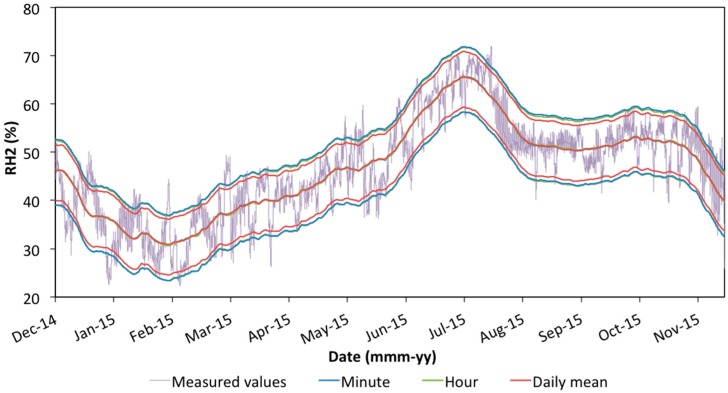
Seasonal RH cycles determined as the 30 days central moving average (MA) of RH data collected by Sensor 2 with different sampling methods. The lower and upper limit bands are calculated as the MA plus and minus the 7th and the 93rd percentiles of the short fluctuations, respectively. The grey line represents the RH values measured every minute from 1 December 2014 to 30 November 2015.

Application of the UNI 10829:1999 to data sampled with different methods led to the conclusion that hourly sampling is sufficient to effectively evaluate the ranges of T and RH of the environment under study. In addition to the hourly sampling, the daily means calculated from measurements based on hourly sampling were tested to minimize the quantity of data to work. The historic microclimate was established following the indications reported in European Standard EN 15757:2010 [18]. The 30 days central moving average and the bands of tolerance are shown in Figure 5. The indications of the standard were applied to data with frequencies of one minute and of one hour and to the daily means calculated from one-hour extractions. The results were compared to evaluate the adequacy of the daily averages to replace data of higher frequencies. In the interpretation of the outcomes, measured values that fall below the lower limit band represent a microclimate too dry for hygroscopic artefacts and can be related to the heating system operating in the museum, while collected data found above the upper limit band can be associated with additional sources of moisture such as the opening of windows or the presence of a high number of people standing in the room, etc.

In the following analysis, only data acquired by RH sensors 2 and 4 were used, as the RH fluctuations within the microclimate frames are too small to be studied with the standard. In fact, since the methodology was not conceived for situations like this, the standard considers the calculated limit unnecessarily strict when fluctuations depart by less than 10% from seasonal RH level [18]. As mentioned previously, the period taken into account includes two months associated with an HVAC system malfunction, which represents an abnormal situation for the environment. Even if it is not valid to assess the real historic microclimate of the room of the museum, RH data from this period was used with the purpose of comparing the outcomes given by different sampling methods.

Figure 5 shows the limit bands obtained from minute and hourly sampling data and from the daily mean dataset.

From Figure 5 it is evident that the limit bands calculated from one-minute sampling and from hourly sampling fully overlap, thus demonstrating once again that for this environment there is no need to set the data acquisition time to a frequency higher than one hour. The bands obtained with the daily means, however, lay closer to the MA as a consequence of the operation of averaging that smooths the variations within the same day. The exclusion of 14% of the largest fluctuations contributes to the curves behavior.

Since the same thermo-hygrometric probes are still monitoring the pictures after their relocation, data collected data collected every minute in the new situation from 14 February to 26 March 2016 (42 days in total) were used to test the different sampling conditions, also in the evaluation of the recently changed conservation conditions. In fact, the regulation states that the historical climate to which the artefact has become acclimatized should be kept unchanged when the condition of objects made of organic hygroscopic materials, kept in a specific microclimate for a prolonged period (at least one year), has been found satisfactory [18]. The performance index was here calculated as the percentage of RH values that are maintained inside the acceptable bands of tolerance determined from each dataset following the EN 15757:2010 standard.

Table 7 show the Performance Index results obtained from data collected by the outside sensors (probes 2 and 4) with the frequencies of one minute and one hour and from the daily means.

The PI values in Table 6 show that the RH data collected in the new environment fall within the safe variability band only in 62.5% of cases for sensor 2 and in 59.8% of cases for sensor 4. Furthermore, as the period taken into account lies in a part of the RH series that is not affected by anomalies, these results can be considered valid also for the microclimatic evaluation. By comparing the outcomes, the hourly sampling proved to be enough to achieve the same results as those given by the measurements every minute. The daily means’ PI values deviate by approximately 10%. However, the period used in the test is very short (less than a month and a half) and it is likely that the results would be in better agreement by repeating the analysis over a longer period of time.

**Figure 6:**
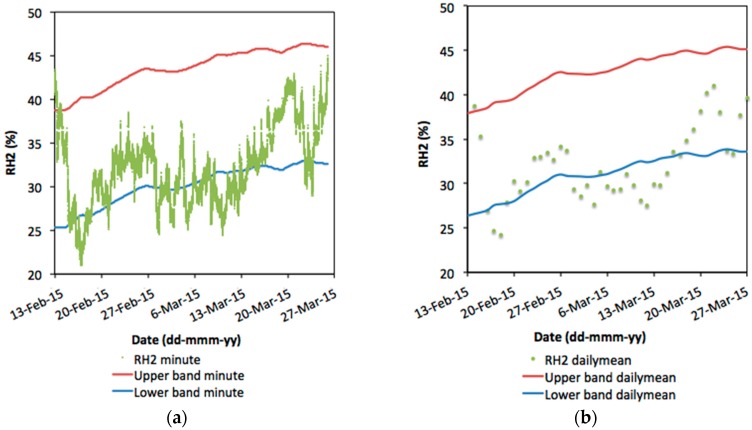
RH data of the 42 days tested (from 14 February 2016 to 26 March 2016) and associated limit bands of one-minute sampling (**a**) and of daily means calculated with the sampling every hour (**b**).

In Figure 6a,b, RH data of the 42 days tested are plotted together with the corresponding limit bands, respectively, in the case of the measurements every minute and of the daily means calculated with the hourly sampling.

The large difference in the amount of data employed in the evaluation is shown clearly. Therefore, the daily mean is much easier to be handled and provides a reasonable approximation of the results in the application of the standard.

### 3.4. ASHRAE Guidelines (2011)

The six climate classes suggested by the ASHRAE [21] for relative humidity have set points of 50% or the historical annual average for permanent collections and between 15 °C and 25 °C for temperature. Table 8 summarizes the percentages of occurrence of both T and RH values that fit together into the specifications of each class.

In the museum room (probes 2 and 4), the PI reached 100% only for class D, which is the least demanding category in terms of thermo-hygrometric control. On the contrary, the quality class of the environment inside the microclimate frames is B, which indicates just a tiny risk to most paintings, most photographs, some artefacts, and some books [21]. 

Table 9a,b summarizes the percentages of data that fit into the specifications of the classes for each physical parameter separately.

From the comparison between Table 9a,b it is evident that temperature is the main driving force in the attribution of ASHRAE classes to the frames’ microclimates. In addition, the environment of the room appears to be threatened by severe fluctuations in relative humidity (see also Figure 4), which lowered the quality of microclimatic control to class D. Inside the microclimate frames, even if the results for T values are the same for both the room and the frames, RH values were maintained extremely stable by the action of the buffer. Therefore, temperature becomes the only factor responsible for the quality of conservation environment. In any case, better conservation conditions could not be achieved due to the malfunctioning HVAC system.

Table 10 and Table 11 show the differences between PI_MINUTE_ reference in Table 8 and the PI values associated with the simulated sampling conditions (ΔPI_HOUR_ and ΔPI_DAILYSPAN_).

The PI values of the combination of T and RH data fitting into the specifications calculated from the hourly sampling and from the daily means are in general agreement with the reference results (Table 8). However, although the differences found with the outcomes of the daily cycles could attenuate the influence of external factors, the PI values obtained do not affect the general attribution of the classes in any case. Likewise, the analysis of separate physical parameters led to the same results as those discussed for Table 9. Nevertheless, it is worth noting that the two microclimates under investigation are quite stable and that in more variable environments the differences between the results could compromise the final evaluation.

## 4. Conclusions

The environment of the museum room is characterized by high variability, both in terms of seasonal and of short-term variations. Organic hygroscopic materials are the most vulnerable materials to deterioration when short term RH fluctuations affect the environment, inducing mechanical damage to objects [3,4,5,18,22] Nevertheless, it was found that the microclimatic frames provided for the Sorolla portraits, maintained stable values of relative humidity and were able to cut off their highest changes also during a malfunctioning in the museum’s HVAC system.

Application of the UNI 10829:1999 to data sampled with different methods led to the conclusion that hourly sampling is sufficient to effectively evaluate the ranges of T and RH of the environment under study. The Italian standard, however, recommends also calculating daily variation of the thermo-hygrometric parameters, which were found to be strongly correlated to the sample rates of measurements. Likewise, in the application of EN 15757:2010 and ASHRAE guidelines of 2011, hourly sampling provided the same outcomes as those with the higher frequency of one-minute measurements. The daily means calculated from the hourly sampling were tested to minimize the quantity of data to work with and were found to be able to guarantee approximate results. The historic microclimate’s limit bands obtained with the daily means lay closer to the MA (because the operation of averaging smooths the variations within the same day) and the calculation of the Performance Index showed that the evaluation of a short set of RH data differs by 10% from the reference result. In application of the ASHRAE guidelines, daily means proved to be able to attribute the class of control that better represents the environment for all the probes.

The best frequency to measure temperature and relative humidity in a controlled indoor environment was found to be of one hour. This rate of acquisition assured reliable results in application of all the standards tested. However, the daily means calculated from the sampling every hour can be considered a valuable tool to achieve a first approximation of compliance with the environmental parameters’ requirements of recent standards for cultural heritage conservation and to quickly evaluate structures with a large number of rooms in their entirety, e.g., detecting situations that need to be investigated further with the hourly datasheet.

For this microclimate, a datalogger working on two files is proposed: one storing hourly data and another storing the related daily means. The exploratory analysis of the daily means allows a swift assessment of conservation risks and a more detailed research into the main causes of problems can be highlighted, where necessary, through the hourly datasheet.

## Figures and Tables

**Table 1:** Position of temperature (T) and relative humidity (RH) probes in the monitoring campaign.

Position	T and RH Probes	
	Painting A	Painting B
Inside	1	3
Outside	2	4

**Table 2:** Daily cycles of T and RH data collected from each probe, expressed in terms of average, maximum and mode of the daily T and RH spans inside (probes 1 and 3) and outside (probes 2 and 4) the microclimate frames.

Probe	Daily Cycles					
	T (°C)			RH (%)		
	Average	Maximum	Mode	Average	Maximum	Mode
1	0.8	3.1	0.5	1.5	5.6	1.5
2	1.3	4.1	1.0	7.9	24.6	6.6
3	0.9	3.2	0.5	1.4	5.7	1.4
4	1.1	3.6	1.0	7.5	27.0	5.9

**Table 3:** Performance Index calculated as the percentage of values of temperature and relative humidity (PI_MINUTE(range)_) that fall inside the range recommended in the UNI 10829:1999 standard for the conservation of objects made of paper.

Probe	PI_MINUTE_ (%) Range	
	18 °C < T < 22 °C	40% < RH < 55%
1	11	100
2	25	53
3	12	77
4	17	52

**Table 4:** Differences between reference PI_MINUTE(range)_ values (Table 3) and PI obtained with datasets associated with extractions every 15 min, every 30 min, and every hour. The Performance Index is defined as the percentage of T and RH values that fall inside the range recommended in the UNI 10829:1999 standard for the conservation of paper. The differences ΔPI_15Min_, ΔPI_30Min_ and ΔPI_Hour_ are calculated as (PI_15Min_ – PI_MINUTE_), (PI_30Min_ – PI_MINUTE_), and (PI_Hour_ – PI_MINUTE_), respectively.

ΔPI (%) Range						
Probe	ΔPI_15Min_ (%)		ΔPI_30Min_ (%)		ΔPI_Hour_ (%)	
	T	RH	T	RH	T	RH
1	0.0	0.1	0.0	0.1	−0.1	0.1
2	0.0	−0.1	0.0	−0.1	0.0	−0.1
3	0.0	0.1	0.0	−0.4	0.0	−0.3
4	−0.1	0.1	−0.1	0.0	0.0	−0.1

**Table 5:** Performance Index calculated as the percentage of values of temperature and relative humidity (PI_MINUTE(daily span)_) that are inside the limit daily span recommended in the UNI 10829:1999 standard for the conservation of objects made of paper.

Probe	PI_MINUTE_ (%) Daily Span	
	ΔT < 1.5°C	ΔRH < 6%
1	91	100
2	74	30
3	89	100
4	82	40

**Table 6:** Differences between reference PI_MINUTE(daily span)_ values (Table 5) and PI obtained with datasets associated with extractions every 15 min, every 30 min and every hour. The Performance Index is defined as the percentage of T and RH daily fluctuations that fall inside the limit daily span recommended in the UNI 10829:1999 standard for the conservation of paper. The differences ΔPI_15Min_, ΔPI_30Min_, and ΔPI_Hour_ are calculated as (PI_15Min_ – PI_MINUTE_), (PI_30Min_ – PI_MINUTE_), and (PI_Hour_ – PI_MINUTE_), respectively.

ΔPI (%) Daily Span						
Probe	ΔPI_15Min_ (%)		ΔPI_30Min_ (%)		ΔPI_Hour_ (%)	
	ΔT	ΔRH	ΔT	ΔRH	ΔT	ΔRH
1	0.5	0.0	0.5	0.0	1.1	0.0
2	1.9	14.8	4.1	18.0	8.2	22.3
3	0.6	0.0	1.4	0.0	3.0	0.0
4	1.7	8.5	2.5	12.1	4.9	14.5

**Table 7:** Performance Index (PI) calculated as the percentage of time in which the RH values of the new environment are maintained inside the acceptable bands of tolerance during the period from 14 February to 26 March 2016. The bands of tolerance have been determined applying the EN 15757:2010 recommendations to datasheets of difference sampling frequency.

Probe	PI (%)		
	Minute	Hour	Daily Mean
2	62.5	62.1	50.0
4	59.8	59.5	50.0

**Table 8:** Performance Index (PI) calculated as the percentage of time for which the combination of T and RH data fits into the specifications of each ASHRAE climate class in the case of one-minute sampling.

Class	PI_MINUTE_ (%)—T and RH Combined			
	Probe 1	Probe 2	Probe 3	Probe 4
AA	97	33	97	33
As	97	59	97	60
A	97	67	97	67
B	100	90	100	90
C	100	99	100	96
D	100	100	100	100

**Table 9:** Performance Index (PI_MINUTE_) of T (**a**) and RH (**b**) values that fit individually into the specifications of each ASHRAE climate class in the case of one-minute sampling.

(a)				
Class	PI_MINUTE_ (%)—Temperature			
	Sensor 1	Sensor 2	Sensor 3	Sensor 4
AA	97	97	97	97
As	97	97	97	97
A	97	97	97	97
B	100	100	100	100
C	100	100	100	100
D	100	100	100	100
**(b)**				
**Class**	**PI_MINUTE_ (%)—Relative Humidity**			
	**Sensor 1**	**Sensor 2**	**Sensor 3**	**Sensor 4**
AA	100	33	100	34
As	100	59	100	60
A	100	67	100	67
B	100	90	100	90
C	100	99	100	96
D	100	100	100	100

**Table 10:** Differences between reference PI_MINUTE_ values in Table 2 and PI obtained with the dataset associated to extractions every hour. Performance Index (PI) is calculated as the percentage of time for which the combination of T and RH data fits into the specifications of each ASHRAE climate class. The differences ΔPI_HOUR_ are calculated as (PI_HOUR_ – PI_MINUTE_).

Class	ΔPI_HOUR_ (%)			
	Probe 1	Probe 2	Probe 3	Probe 4
AA	0.5	−3.1	0.9	−2.8
As	0.5	1.5	0.9	1.8
A	0.5	−4.9	0.9	−3.6
B	0.1	0.0	0.1	0.3
C	0.1	−0.8	0.1	−2.1
D	0.0	0.0	0.0	0.0

**Table 11:** Differences between reference PI_MINUTE_ values in Table 2 and PI obtained with the dataset associated to the daily means. Performance Index (PI) is calculated as the percentage of time for which the combination of T and RH data fits into the specifications of each ASHRAE climate class. The differences ΔPI_DAILYMEAN_ are calculated as (PI_DAILYMEAN_ – PI_MINUTE_).

Class	ΔPI_DAILYMEAN_ (%)			
	Probe 1	Probe 2	Probe 3	Probe 4
AA	1.1	−1.7	0.6	−2.0
As	1.1	7.2	0.6	7.0
A	1.1	1.2	0.6	1.0
B	0.1	1.7	0.1	1.4
C	0.1	−7.3	0.1	−4.6
D	0.0	0.0	0.0	0.0

## Abbreviations

ASHRAE	American Society of Heating, Air-Conditioning and Refrigerating Engineers
EN	European Standard
HVAC	Heating Ventilating and Air-Conditioning
PI	Performance Index (%)
RH	Relative humidity of air (%)
T	Air temperature (°C)
UNI	Ente Nazionale Italiano di Normazione
